# The active form of MMP-3 is a marker of synovial inflammation and cartilage turnover in inflammatory joint diseases

**DOI:** 10.1186/1471-2474-15-93

**Published:** 2014-03-19

**Authors:** Shu Sun, Anne-Christine Bay-Jensen, Morten A Karsdal, Anne Sofie Siebuhr, Qinlong Zheng, Walter P Maksymowych, Thorbjørn G Christiansen, Kim Henriksen

**Affiliations:** 1 Nordic Bioscience Biomarkers and Research, Herlev, Denmark; 2 Nordic Bioscience Biomarkers and Research, Beijing, China; 3 University of Alberta, Edmonton, Canada; 4 Alberta Innovates-Health Solutions, Alberta, Canada; 5 Orthopaedic Surgical Unit, Gentofte University Hospital, Hellerup, Denmark

**Keywords:** MMP-3, Active form, Ankylosing spondylitis, Rheumatoid arthritis, Serum

## Abstract

**Background:**

Matrix metalloproteinase-3 (MMP-3) plays an important role in the pathology of rheumatoid arthritis (RA) and ankylosing spondylitis (AS). Measurement of active MMP-3 in clinical samples could provide information about progression of rheumatoid diseases, and potentially response to treatment. Hence, we aimed to develop a sensitive assay specifically measuring the active form of MMP-3 (act-MMP-3) both in *ex vivo* models and in human sera.

**Methods:**

A monoclonal antibody against the first 6 amino acids of act-MMP-3 was developed, and the specificity was carefully tested by comparing total and active MMP-3. A technically robust act-MMP-3 ELISA was produced. For biological validation, human synovial membrane and human cartilage explant (HEX) culture models were measured and compared by ELISA and immunoblots. For clinical relevance, the serum levels of act-MMP-3 in AS and RA patients before and after anti-TNF-α treatment were evaluated.

**Results:**

A highly specific and technically robust ELISA detecting act-MMP-3 in serum was developed. The lower limit of detection was 33.7 pg/mL. The dilution and spiking recovery of human serum was within 100 ± 20%. The average intra- and inter-assay variations were 3.1% and 13.5% respectively.

High levels of act-MMP-3 expression were observed in human synovial membrane culture and oncostatin M and TNF-α stimulated human cartilage. In a cross-sectional study of both AS and RA patients, serum act-MMP-3 level was correlated with C-reactive protein (CRP) and erythrocyte sedimentation rate (ESR). In addition, in patients receiving anti-TNF-α treatment, the serum level of act-MMP-3 was significantly reduced compared to baseline level reflecting the anti-inflammatory effects of the treatment.

**Conclusion:**

We have successfully developed an assay measuring act-MMP-3 in human serum showing correlation to inflammatory markers. Further studies are required to clarify, whether act-MMP-3 can serve as a predictive marker for outcome in chronic rheumatoid disorders.

## Background

Ankylosing spondylitis (AS) is a chronic inflammatory rheumatic disease, which primarily affects the spine and sacroiliac joints [1]. Rheumatoid arthritis (RA) is another chronic autoimmune disease with progressive synovial joint destruction. Increased turnover of extracellular matrix (ECM) proteins has been found in both AS and RA, and the MMPs are known to be important proteases responsible for ECM protein degradation [2]. Previous studies have indicated MMP-3 as a pathological mediator in AS, as well as RA [3,4].

The pathology of RA includes different cells of the joint such as chondrocytes, T cells, fibroblast-like synoviocytes and macrophages, features which also are seen in AS. Among them, fibroblast-like synoviocytes are thought to be key players in RA [5,6], and they are known to express MMP-3, which is linked to their ability to cleave aggrecan, collagen type II, IX, X, link proteins and others in the joint [7,8]. In addition, MMP-3 can activate other MMPs, such as pro-MMP-1, pro-MMP-8, pro-MMP-9 and pro-MMP-13 [9-12], and hence MMP-3 is considered an important pathological mediator of AS and RA.

MMP-3 is composed of a signal peptide, which is cleaved off during the secretion process, a pro-domain, which is cleaved during the activation process, a catalytic domain which has a conserved zinc-binding site, a hinge domain, and a hemopexin domain [13]. Pro-MMP-3 is secreted to the ECM in its latent form and is then activated by removal of the pro-peptide, which is mediated by serine proteases, plasmin and trypsin [14,15].

Serum levels of MMP-3 have been reported elevated in RA patients compared with osteoarthritis patients [16], and to be associated with the development of structural damage in RA patients [17]. Furthermore, serum MMP-3 levels at baseline were shown to be predictive of radiographic progression in an RA cohort [18]. In AS, MMP-3 was also found to be a predictor of structural progression [19]. Collectively, the line of data underscores the pathological relevance of MMP-3 in arthritic diseases. In addition, elevated MMP-3 expression has been observed in isolated synovium and cartilage [20,21].

Presently, two methods are used to measure serum levels of MMP-3, and these measure either total MMP-3 protein [22] or the activity of MMP-3 using a substrate; however, neither of these approaches provides specific information about the active form of MMP-3. It is known that the cleavage capacity of MMP-3 is regulated by three levels: synthesis, activation and inhibition. Only act-MMP-3 has the ability to cleave ECM proteins, while the concentration of latent form only reflects the potential of MMP-3 to degrade proteins. So act-MMP3 may provide other information beyond total MMP-3, like the activation of pro-MMP3, and the abnormal imbalanced binding between act-MMP3 and inhibitors in rheumatoid diseases. Moreover, it is also useful in drug screening process which can show the treatment efficiency on reducing active protease. Since pro-MMP3 concentration is much higher than act-MMP3 in biological fluid, this information may have been masked by total MMP3 measurement. So in this study we focused on developing an ELISA specifically detecting active MMP-3, and we characterized it using both *ex vivo* cultures of human cartilage and synovium, and serum samples from AS and RA cohorts.

## Methods

### Reagents

All the reagents used in this study were standard high quality chemicals from Sigma (St.Louis, MO, USA) and Merck (Whitehouse Station, NJ, USA) unless specifically mentioned. All the peptides for monoclonal antibody development were a) immunogenic peptide: FRTFPGIPKW-GGC b) screening peptide: FRTFPGIPKW-biotin c) standard peptide: FRTFPGIPKW d) elongated peptide: HFRTFPGIPKW. All the peptides were purchased from the Chinese Peptide Company, China.

### Development of monoclonal antibody

All the mice were specific pathogen free (SPF) animals and housed in SPF animal facility with 12 h light/dark cycle. The mice had free access to food and water. All the work on mice was approved by Beijing laboratory animal administration office and animal ethics committee of Nordic Bioscience (Beijing).

We used the first 10 amino acids of the N-terminal (^100^’FRTFPGIPKW’^109^) as the immunogenic peptide to generate specific neo-epitope monoclonal antibodies. The methods used for monoclonal antibody development were as previously described [23]. Briefly, six Balb/c mice (female, 4 to 6 weeks old) were immunized subcutaneously with 200 μl emulsified antigen and 60 μg of KLH conjugated immunogenic peptide. Consecutive immunizations were performed at two-week intervals in Freund's incomplete adjuvant, until stable sera titer levels were reached, and the mice were bled from the 3rd immunization on. At each bleeding, the serum titer was detected and the mouse with highest antiserum titer and the best native reactivity was selected for fusion. The selected mouse was rested for 1 month followed by intravenous boosting with 50 μg of KLH conjugated immunogenic peptide in 100 μl 0.9% sodium chloride solution 3 days before isolation of the spleen for cell fusion.

The fusion procedure has been described [24]. Briefly, the spleen cells from the immunized mouse with best antiserum titer and native reactivity were fused with SP2/0 myeloma fusion partner cells. The fusion cells were raised in 96-well plates and incubated in a 5% CO2 incubator. Here standard limited dilution was used to promote monoclonal growth. After seven to ten days of culture, supernatants were screened in a competitive ELISA setting. Cell lines specific to standard peptide and without cross-reactivity to elongated peptide were selected and sub-cloned. At last the antibodies were purified.

### In vitro Activation of MMP-3

10 μg of Pro-MMP-3 (cat.no PF063, Calbiochem) was dissolved in 100 μL MMP buffer (100 mM Tris-HCl, 100 mM NaCl, 10 mM CaCl_2_, 2 mM Zn acetate, pH 8.0). 1 μg pro-MMP-3 was mixed with 1.1 μL 10 mM APMA and incubated at 37°C for 3 hours.

### *Ex vivo* Synovial membrane tissue culture

Synovial membrane was obtained from total knee replacements of osteoarthritis patients at Gentofte Hospital, Gentofte, Denmark. The study was approved by the Ethics Committee of the Capital Region of Denmark, DK-3400 (approval no. HD-2007-0084). Patients were informed about the purpose of the study and provided written consent.

Synovial membrane was isolated during surgery and kept in DMEM + 10% FCS at 4°C until the next day, where experiments were initiated. Synovial membrane was washed 5 times in PBS to limit contamination and to remove excess blood. The synovial membrane was divided into equal pieces (explants) of about 30 mg and placed in a 96 well plate. Explants for the metabolic inactive (MI) group were inactivated by three freeze–thaw cycles. They were placed in a 37°C water bath for 5 min, then immediately transferred to liquid nitrogen and stayed for another 5 min, and repeated for 3 times. The explants was cultured in DMEM:F12 for 14 days, where medium was changed every second or third day. Conditioned medium was kept at -20°C until analysis.

### Human cartilage explants (HEX) culture

The human cartilage explants (HEX) culture was performed as described previously [25]. Briefly, human cartilage was collected from cartilage replacement surgery. The study was approved by the Ethics Committee of the Capital Region of Denmark, DK-3400 (approval no. HD-2007-0084). The cartilage explants (16 ± 4 mg) were placed in 96-well plates and cultured at 37°C, 5% CO2 incubator. Each explant was cultured in 200 μL of DMEM for 21 days and divided into two groups: 1) without catabolic factors (W/O), 2) with the catabolic cytokines oncostatin M (10 ng/mL) and TNF-α (20 ng/mL) (O + T) to stimulate MMP activity. The culture medium was changed every 2-3 days and the supernatant was collected and stored in -20°C for further use.

### Western blotting

Synovial membrane culture supernatant, HEX supernatant and act-MMP-3 were separated by SDS-PAGE, and proteins were transferred onto nitrocellulose membranes. The membranes were blocked with 5% skim milk powder in TBS-T buffer, and incubated in room temperature for 2 hours. The membranes were incubated with primary antibody at 4°C overnight, followed by three times wash in TBS-T buffer. Then the membranes were incubated in the secondary peroxidase conjugated antibody, and followed by three times wash. Finally, the results were visualized with ECL detection system (cat# RPN2109, Amersham Pharmacia). The antibody recognizes both pro and active form of MMP-3 was from Abcam (ab38916).

### Active MMP-3 assay protocol

ELISA-plates used for the assay development were Streptavidin-coated from Roche cat.: 11940279. All ELISA plates were analyzed with ELISA reader from Molecular Devices, SpectraMax M, (CA, USA). The selected monoclonal antibody 7B2 was labeled with horseradish peroxidase (HRP) using the Lightning link HRP labeling kit according to the instructions of the manufacturer (Innovabioscience, Babraham, Cambridge, UK). A 96-well streptavidin plate was coated with screening peptide FRTFPGIPKW-biotin dissolved in coating buffer (50 mM Tris, 137 mM NaCl, 1% BSA, 0.05% Tween-20, 0.36% Bronidox L5, pH 8.0) and incubated 30 minutes at 20°C. 30 μL of standard peptide or sample dissolved in incubation buffer (50 mM Tris, 137 mM NaCl, 1% BSA, 0.05% Tween-20, 0.36% Bronidox L5, 5% liquid II, pH 8.0) were added to appropriate wells, and followed by 100 μL of conjugated antibody 7B2-HRP. Synovial membrane culture and HEX culture supernatant, serum samples measured in the ELISA were pre-diluted 1:2; act-MMP-3 and pro-MMP-3 inhibition test was performed in a serial dilution with the concentration from 1 ng/ul. Then the assay was allowed to incubate at 4°C for 20 ± 1 hours. Finally, 100 μL tetramethylbenzinidine (TMB sens) (Kem-En-Tec cat.4850E) was added and the plate was incubated 15 minutes at 20°C in the dark. All the above incubation steps included shaking at 300 rpm. After each incubation step the plate was washed five times in washing buffer (20 mM Tris, 50 mM NaCl, pH 7.2). The TMB reaction was stopped by adding 100 μL of stopping solution (0.1% H_2_SO₄) and measured at 450 nm with 650 nm as the reference. A standard curve was performed by serial dilution of the standard peptide with the concentration of 0, 4.88, 9.77, 19.53, 39.06, 78.13, 156.25, 312.5, 625, 1250, 2500 and 5000 pg/mL. Calibration curve was plotted using a 4-parametric mathematical fit model.

### Technical evaluation

The intra- and inter-assay variation was determined by 10 independent runs of 8 quality control (QC) samples consisting of serum, and each run consisting of double determinations of the samples. The lower limit of detection (LLOD) was determined from 21 zero samples (incubation buffer) and calculated as the mean 3x standard deviation. Dilution recovery was calculated as a percentage of recovery of diluted QC samples from the 100% sample. Spike recovery was calculated by comparing different concentrations of human cartilage explants supernatant in incubation buffer and in human serum. Finally, for each assay, a master calibrator prepared from synthetic peptides accurately quantified by amino acid analysis was used for calibration purposes.

### Clinical samples

Serum samples from 201 AS patients and 47 control RA patients were analyzed [26]. Of all the patients, 142 AS patients and 47 RA patients received 3 months anti-TNF-α treatment after recruitment. At baseline, the Bath AS Disease Activity Index (BASDAI) and the modified Stoke AS Spine Score (mSASSS) were recorded for all the AS patients, while DAS and HAQ were recorded for RA patients. CRP (C-reactive protein) concentration and ESR (erythrocyte sedimentation rate) were also recorded for both AS and RA patients. For those patients who have received anti-TNF-α drug, the characteristics mentioned above, except for mSASSS, were also recorded after treatment. In 118 AS patients, mSASSS were recorded after 2 years of followup. All the clinical characteristics used have previously been published by Bay-Jensen AC *et al*[26]. Serum samples were thawed from -80°C storage and act-MMP-3 concentrations were measured both at baseline and after treatment. The study was approved by local ethics committee and all patients provided the written informed consent.

### Statistics

Mean values and standard error of the mean (SEM) were calculated using GraphPad Prism, version 6 (GraphPad Software, San Diego, CA, USA). Act-MMP-3 differences between female and male were analyzed using non-parametric Mann–Whitney test. Correlations between act-MMP-3 and patient clinical characteristics (age, disease duration, CRP, ESR, BASDAI, mSASSS) were determined by Spearman’s test. Significant differences of clinical samples between pre-treatment and post-treatment were determined using the Wilcoxon matched-pairs signed rank test. Significant differences were considered if P < 0.05.

## Results

### Assessment of antibody performance using the pro and active form MMP-3

**Figure 1:**
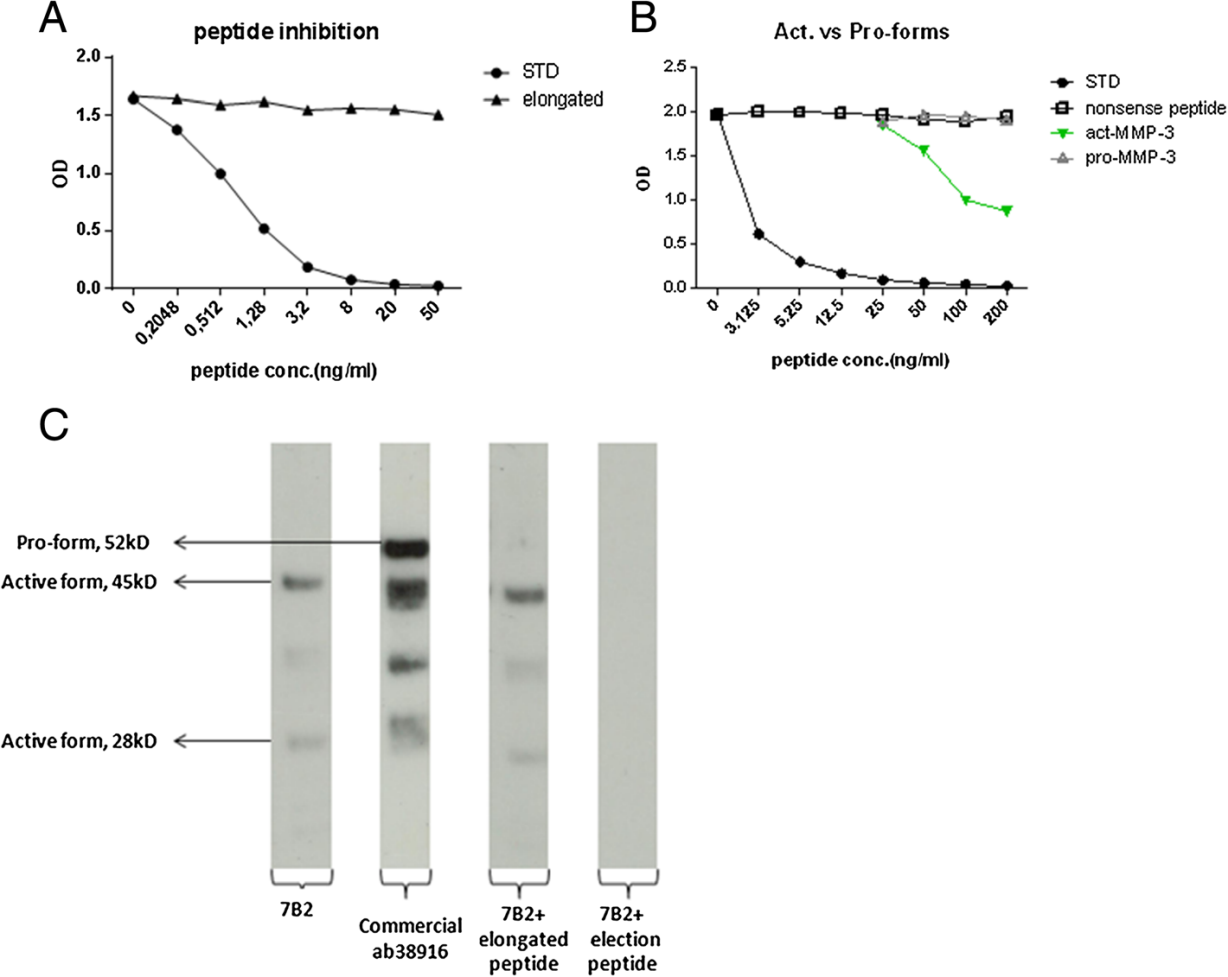
**7B2 has specific reactivity towards act-MMP-3. (A)** Reaction towards to standard peptide and elongated peptide of selected monoclonal antibody 7B2. **(B)** Competitive ELISA results of act-MMP-3 and pro-MMP-3. **(C)** Western blot results of in vitro activated MMP-3 using 7B2 as primary antibody.

When measured in the competitive act-MMP-3 assay applying the 7B2 antibody, the antibody specifically recognized the N-terminal peptide ^100^’FRTFPGIPKW’^109^ and activated MMP-3 but did not recognize the elongated peptide ^99^’HFRTFPGIPKW’^109^ and pro-MMP-3 (Figure 1A,B). The results were confirmed by western blot of in vitro activated MMP-3. Bands were detected at 45 kD and 28 kD, but not at 52 kD, which is the size of the pro-MMP-3. All three bands were detected by the antibody recognizing the total MMP-3 antibody (Figure 1C). To ensure the specificity of the blot, primary antibody incubation was done including either the elongated peptide or the standard peptide. The signal was completely blocked only in the presence of the standard peptide.

### Technical performance of the act-MMP-3 assay

The LLOD of the ELISA was 33.7 pg/mL. The average intra- and inter-assay variations were 3.1% and 13.5%, respectively. The dilution and spiking recovery of human serum were within 100 ± 20% (Table 1). The data confirms technically robustness of the assay.

**Table 1:** Technical performance of act-MMP-3 assay

**Measurements**	**Technical characteristics**
Lower limit of detection	33.7 pg/mL
Intra-assay variability	3.1%
Inter-assay variability	13.5%
Dilution recovery	Within 100 ± 20%
Spiking recovery	Within 100 ± 20%

### Assessment of antibody performance using material from human synovial membrane and cartilage *ex vivo* cultures

**Figure 2:**
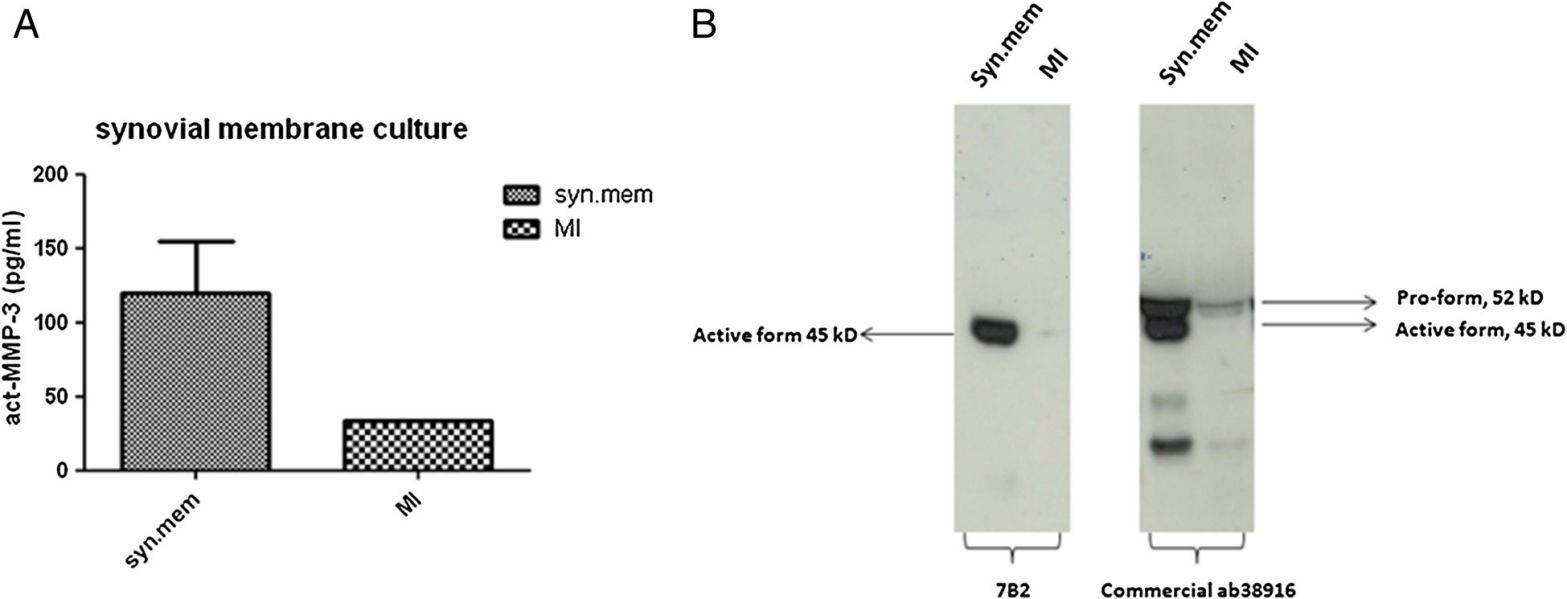
**Act-MMP-3 profile in human synovial membrane culture. (A)** Act-MMP-3 expression in synovial membrane culture supernatant. **(B)** Western blot results of synovial membrane culture.

We investigated the act-MMP-3 expression and release in human synovial membrane and HEX cultures, and as expected act-MMP-3 was expressed by human synovial membrane, compared to the negative control MI group (Figure 2A). The data was confirmed by western blotting, which again confirmed that the antibody only recognized the active form of MMP-3 (Figure 2B).

In HEX culture, stimulation with the pro-inflammatory cytokines TNF-α and oncostatin M resulted in the release of act-MMP-3 into culture medium starting from the early culture stage (see Additional file 1: Figure S1), supporting the pathological relevance of act-MMP-3 in inflammatory joint diseases.

### Act-MMP-3 levels in serum are related to inflammatory status in AS and RA

We investigated the association between baseline act-MMP-3 levels and age, gender, disease duration in AS patients. The data showed that age and disease duration were not correlated to act-MMP-3 levels (R = -0.046 and R = -0.032, respectively). Act-MMP-3 levels in male and female patients were not significantly different. We also assessed the correlation between baseline act-MMP-3 levels, baseline mSASSS, baseline BASDAI, baseline CRP and baseline ESR in AS patients (Table 2). Act-MMP-3 levels were not correlated with the disease burden markers, mSASSS and BASDAI (R = 0.10 and R = 0.06, respectively), but were significantly correlated to CRP and ESR (R = 0.42 and R = 0.38, respectively, P < 0.001), which are markers of inflammation. Similar data were obtained in samples from RA patients, with baseline serum act-MMP-3 levels significantly correlated to CRP, but with no correlation with disease activity score (DAS) and health assessment questionnaire score (HAQ) (see Additional file 1: Table S1).

**Table 2:** Univariate correlation between clinical parameters and serum act-MMP-3 level in AS patients

	**Baseline active MMP3 (pg/ml)**		
	**R**	**P**	**N**
Age (years)	-0.05	0.55	173
Disease duration (years)	-0.03	0.68	168
CRP (mg/dl)	0.42	<0.0001	167
ESR (mm/hour)	0.38	<0.0001	171
Baseline BASDAI	0.06	0.46	171
Baseline mSASSS	0.10	0.25	130

### Baseline act-MMP-3 levels predict anti-inflammatory response to anti-TNF-α treatment

To evaluate if baseline act-MMP-3 level had any predictive value response to anti-TNF-α treatment, correlations between baseline act-MMP-3 level, BASDAI reduction (change < 0), CRP reduction (change < 0) and ESR reduction (change < 0) in AS patients were analyzed (Table 3). The data showed that baseline act-MMP-3 levels were significantly and positively correlated to the reduction in CRP and ESR (R = 0.44 and R = 0.39, respectively, P < 0.001). There were no significant correlations between baseline act-MMP-3 level and BASDAI reduction (R = 0.10). Comparable results were observed for the RA patients. Baseline act-MMP-3 level did not predict DAS and HAQ change, but were correlated to CRP and ESR changes (see Additional file 1: Table S2).

**Table 3:** Univariate correlation between clinical characteristics change after treatment and serum act-MMP-3 level in AS patients

	**Baseline active MMP-3 (pg/ml)**		
	**R**	**P**	**N**
CRP	0.44	<0.0001	107
Reduction (mg/dl)			
ESR	0.39	<0.0001	113
Reduction (mm/hour)			
BASDAI reduction	0.10	0.26	120

To investigate if act-MMP-3 level can predict 2-year radiographic progression, correlation between baseline act-MMP-3 level and 2-year mSASSS increase (change > 0) were analyzed. There was no significant correlation between baseline act-MMP-3 level and 2-year mSASSS change (R = 0.07).

### Serum levels of act-MMP-3 are reduced by anti-TNF-α treatment

**Figure 3:**
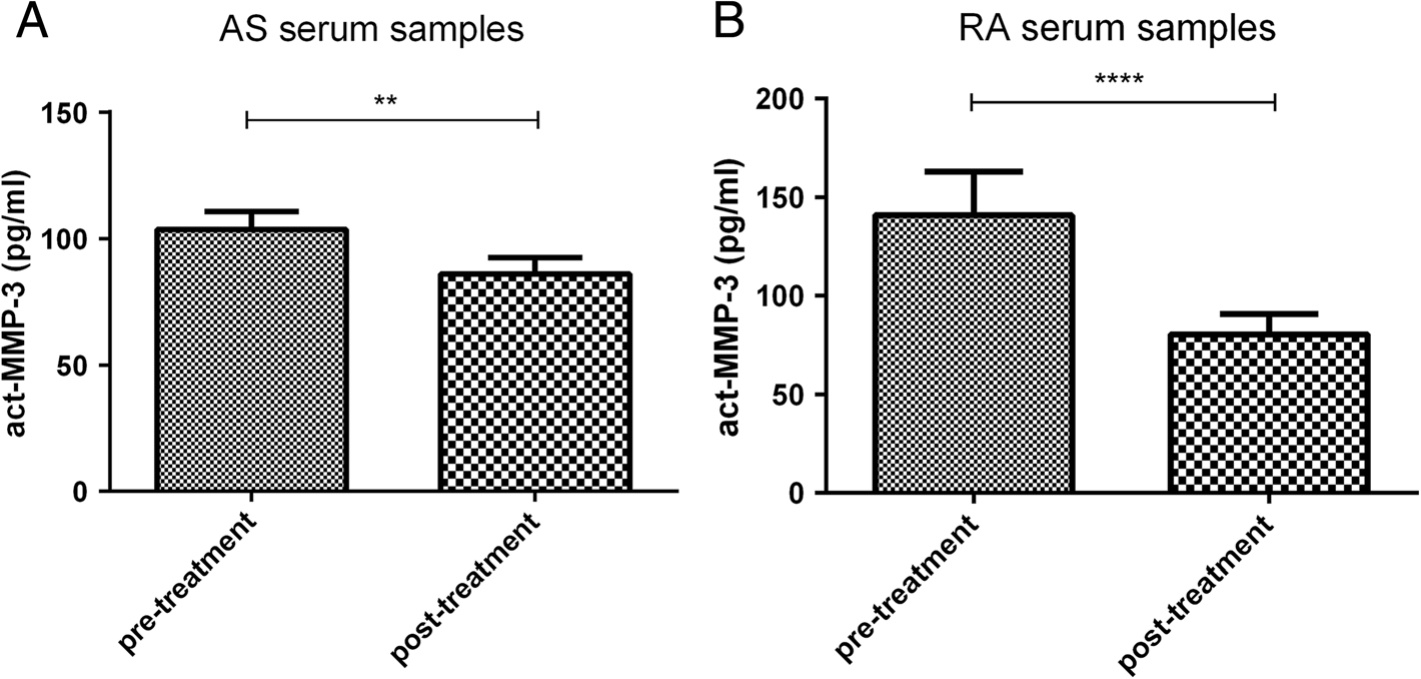
**Act-MMP-3 assay measured in AS and RA patients at baseline and after treatment.** The concentration of act-MMP-3 significant decreased after anti-TNF-α treatment in **(A)** AS patients (n = 98) and **(B)** RA patients (n = 41). (** = P < 0.01, **** = P < 0.0001).

To investigate if act-MMP-3 levels responded to anti-TNF-α treatment, we measured serum levels of act-MMP-3 in AS and RA patients at baseline and after anti-TNF-α treatment. A significant decrease in serum act-MMP-3 after treatment (Figure 3) was observed for both AS and RA, which showed the anti-TNF-α treatment, could effectively reduce act-MMP-3 levels, which correlates well with the previous findings that act-MMP-3 reflects the inflammatory status of the patients.

## Discussion

MMP-3 is considered an important protease in joint damage where it has been shown to cleave a series of ECM proteins [7,8]. Monitoring act-MMP-3 levels is limited by the present commercial assays, which cannot distinguish the pro-form and act-form MMP-3. This illustrates a need for an assay that specifically measures act-MMP-3, and which can be used in *ex vivo* models and in clinical samples.

In this study, we successfully produced a monoclonal antibody specifically recognizing act-MMP-3, while no cross-reactivity to an elongated peptide with one more amino acid at the N-terminal. Additionally, the antibody only detected act-MMP-3 and not pro-MMP-3, clearly confirming the specificity of the antibody. Furthermore, the antibody recognized both forms of act-MMP-3, namely the 45 kD and 28 kD forms, as expected since the low Mw form is generated by a C-terminal truncation [14,27].

We applied two *ex vivo* models to clarify the pathological relevance of MMP-3 expression. As expected from literature [28,29], we found high levels of act-MMP-3 in both synovial membrane culture and in Oncostatin M and TNF-α stimulated human cartilage explants, when compared to controls.

In RA and AS patients, we successfully measured act-MMP-3, and we found the concentration significantly decreased after anti-TNF-α treatment, which could indicate that act-MMP-3 could be used as a predictor of the treatment efficiency at the level of anti-inflammatory effects, whereas the levels did not correlate to or predict changes in disease activity scores. Furthermore, act-MMP-3 significantly correlated with CRP and ESR levels, which showed act-MMP-3 is a marker of inflammation. In previous studies, CRP, ESR correlated to mSASSS or BASDAI in AS patients [4,19] and CRP weakly correlated to 2-years’ mSASSS change [19], which indicate act-MMP-3 behaves different than the classical inflammatory markers.

In a previous study, serum MMP-3 could predict 2-year mSASSS change [19]; however, we did not reproduce this data, which could be explained by two reasons. Firstly, the AS patients in the previous study did not receive anti-TNF-α treatment. Secondly, an MMP-3 assay which measured both pro-MMP-3 and act-MMP-3 was used in the previous study, while in our study we only measured act-MMP-3. We found act-MMP-3 only correlated with the inflammation markers CRP and ESR, but not to other measures of disease burden in AS (BASDAI, mSASSS) and RA (DAS, HAQ), and hence it appears that act-MMP-3 reflects other aspects of disease than total MMP-3.

However, there are some limitations to consider in relation to the detection of act-MMP-3 in human serum. Active forms of MMPs are known to bind to carrier proteins like TIMP and α-2 macroglobulin (α_2_M) which are abundant in biological fluid [30,31]. α_2_M is a tetramers assembled by four 180 kD subunits, which can capture act-MMPs and shield them in the subunits [32], and this could skew the levels in serum towards lower levels. Furthermore, the interaction between α_2_M and act-MMP-3 is covalent and thereby virtually impossible to break without destroying antigen-antibody reaction. This could be the explanation for the low concentrations of free act-MMP-3 detected in human serum. Importantly, even at very low levels act-MMP-3 still has pathological relevance.

## Conclusions

In summary, we have developed a highly sensitive and reproducible assay monitoring act-MMP-3 level in serum from humans. In *ex vivo* models, we confirmed act-MMP-3 can be expressed by synovial membrane and oncostatin M and TNF-α stimulated human cartilage. Furthermore, we found act-MMP-3 in human serum showing correlation to inflammatory markers. Further studies are required to clarify, whether act-MMP-3 can serve as a predictive marker for outcome in chronic rheumatoid disorders.

## Abbreviations

Act-MMP-3: Active matrix metalloproteinase-3; APMA: 4-aminophenylmercuric acetate; AS: Ankylosing spondylitis; BASDAI: Bath ankylosing spondylitis disease activity index; CRP: C-reactive protein; DAS: Disease activity score; ECM: Extracellular matrix; ESR: Erythrocyte sedimentation rate; HAQ: Health assessment questionnaire; HEX: Human cartilage explants culture; LLOD: Lower limit of detection; MI: Metabolic inactive; mSASSS: Modified stoke ankylosing spondylitis spinal score; QC: Quality control; RA: Rheumatoid arthritis; SEM: Standard error of the mean; α2M: α-2 macroglobulin.

## Competing interests

SS, ACBJ, MK, ASS, QLZ and KH are full time employees at Nordic Bioscience. Morten Karsdal holds stock in Nordic Bioscience. None of the authors received fees, bonuses or other benefits for the work described in the manuscript. Other authors have no competing interests.

## Authors’ contributions

SS did the study design, performed most of the experiments, data analysis and wrote the manuscript. KH, ACBJ and MK assisted in study design and data analysis. ASS did the *ex vivo* human synovial membrane culture. WPM provided the clinical samples. TC provided the human synovial membrane samples. QLZ assisted in antibody development. All authors have read and approved the final manuscript.

## Pre-publication history

The pre-publication history for this paper can be accessed here:

http://www.biomedcentral.com/1471-2474/15/93/prepub

## Supplementary Material

Additional file 1 **Includes one figure and two tables. Figure S1** is act-MMP-3 measurement in the supernatant of HEX culture. **Table S1** is the univariate correlation between clinical parameters and serum act-MMP3 level in RA patients. **Table S2** is the univariate correlation between clinical characteristics change after treatment and serum act-MMP-3 level in RA patients. Click here for file
