# Supplementary material for: The active form of MMP-3 is a marker of synovial inflammation and cartilage turnover in inflammatory joint diseases
Source: BMC Musculoskelet Disord. 2014 Mar 19;15:93. doi: 10.1186/1471-2474-15-93 (PMC4003863; doi:10.1186/1471-2474-15-93)
Supplement: Additional file 1 — Includes one figure and two tables. Figure S1 is act-MMP-3 measurement in the supernatant of HEX culture. Table S1 is the univariate correlation between clinical parameters and serum act-MMP3 level in RA patients. Table S2 is the univariate correlation between clinical characteristics change after treatment and serum act-MMP-3 level in RA patients. [file 1471-2474-15-93-S1.docx]

**Supplementary data**


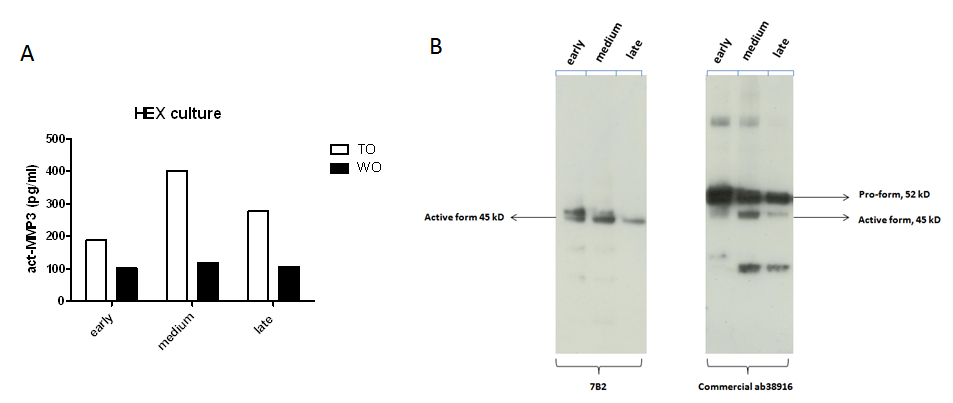


**Figure S1: Act-MMP-3 expression in HEX culture.** The supernatant were collected from the early, medium and late stage of culture. The induction of TNF-α and oncostatin M resulted in the release of act-MMP-3 into culture medium from the early culture stage. Western blot result showed 7B2 only recognized the 45kD active form, while commercial ab38916 antibody recognized both pro and active form.

**Table S1: Univariate correlation between clinical parameters and serum act-MMP3 level in RA patients**

|  | Baseline active MMP3 (pg/ml) | | |
| --- | --- | --- | --- |
|  | R | P | N |
| Age (years) | 0.09 | 0.56 | 47 |
| Disease duration (years) | -0.03 | 0.85 | 41 |
| CRP (mg/dl) | 0.35 | 0.03 | 45 |
| ESR (mm/hour) | 0.22 | 0.17 | 45 |
| DAS | 0.28 | 0.12 | 36 |
| HAQ | 0.14 | 0.40 | 43 |

**Table S2: Univariate correlation between clinical characteristics change after treatment and serum act-MMP-3 level in RA patients**

|  | Baseline active MMP-3 (pg/ml) | | |
| --- | --- | --- | --- |
|  | R | P | N |
| CRP  Reduction (mg/dl) | 0.43 | 0.007 | 44 |
| ESR  reduction (mm/hour) | 0.34 | 0.04 | 44 |
| DAS reduction | 0.15 | 0.41 | 36 |
| HAQ reduction | 0.25 | 0.14 | 42 |
